# Benefits of pallidal stimulation in dystonia are linked to cerebellar volume and cortical inhibition

**DOI:** 10.1038/s41598-018-34880-z

**Published:** 2018-11-21

**Authors:** Anna Fečíková, Robert Jech, Václav Čejka, Václav Čapek, Daniela Šťastná, Ivana Štětkářová, Karsten Mueller, Matthias L. Schroeter, Filip Růžička, Dušan Urgošík

**Affiliations:** 1 Department of Neurology and Centre of Clinical Neuroscience, First Faculty of Medicine and General University Hospital, Charles University, Prague, Czech Republic; 2 Faculty of Biomedical Engineering, Czech Technical University in Prague, Prague, Czech Republic; 3 Department of Neurosurgery, Na Homolce Hospital, Prague, Czech Republic; 4 Department of Neurology, Third Faculty of Medicine, Charles University and Faculty Hospital Kralovske Vinohrady, Prague, Czech Republic; 5 Max Planck Institute for Human Cognitive and Brain Sciences, Leipzig, Germany; 6 Clinic for Cognitive Neurology, University Hospital, Leipzig, Germany; 7 Department of Stereotactic and Radiation Neurosurgery, Na Homolce Hospital, Prague, Czech Republic

## Abstract

Clinical benefits of pallidal deep brain stimulation (GPi DBS) in dystonia increase relatively slowly suggesting slow plastic processes in the motor network. Twenty-two patients with dystonia of various distribution and etiology treated by chronic GPi DBS and 22 healthy subjects were examined for short-latency intracortical inhibition of the motor cortex elicited by paired transcranial magnetic stimulation. The relationships between grey matter volume and intracortical inhibition considering the long-term clinical outcome and states of the GPi DBS were analysed. The acute effects of GPi DBS were associated with a shortening of the motor response whereas the grey matter of chronically treated patients with a better clinical outcome showed hypertrophy of the supplementary motor area and cerebellar vermis. In addition, the volume of the cerebellar hemispheres of patients correlated with the improvement of intracortical inhibition which was generally less effective in patients than in controls regardless of the DBS states. Importantly, good responders to GPi DBS showed a similar level of short-latency intracortical inhibition in the motor cortex as healthy controls whereas non-responders were unable to increase it. All these results support the multilevel impact of effective DBS on the motor networks in dystonia and suggest potential biomarkers of responsiveness to this treatment.

## Introduction

Deep brain stimulation of the globus pallidus internus (GPi DBS) is an effective symptomatic treatment for pharmacoresistant dystonic syndromes^1–3^, where pathophysiological mechanisms of action are not yet fully understood. In contrast to the almost immediate effects of brain stimulation in Parkinson’s disease, DBS in dystonia usually takes several weeks or months to achieve clinically significant improvement^4^ suggesting the induction of long-term plasticity changes responsible for the delayed effect of DBS. The effects of GPi DBS may propagate along anatomical projections and, besides acute impact on the immediate state of the motor network, it may initiate chronic “hardwire” rebuilding in distant cortical and subcortical regions and in the cerebellum. However, the variability of clinical improvement of dystonia after GPi DBS still remains very high^2,5^ with only a few favorable factors derived from the dystonia phenotype, age of onset, GPi volume or electrophysiology observations^6–9^.

Cortical and subcortical motor networks show abnormalities in dystonia with a severe impact on brain electrophysiology and morphology. Several mechanisms have been discussed, describing an alteration of inhibitory circuits at the cortical, brainstem and spinal level^10,11^ or reporting impaired sensorimotor integration^12,13^. These functional changes in dystonia are accompanied by various structural abnormalities^14^ especially in brain regions including the primary sensorimotor cortex, basal ganglia, thalamus and cerebellum^14–17^.

As GPi DBS has a positive influence on motor control in dystonic patients, some impact on the function or structure of the sensorimotor network may be expected. While short-term modulatory effects of GPi DBS may influence the excitability in the sensorimotor network, long-term changes may involve grey matter changes at the cortical and subcortical level. In our study, we compared dystonia patients and healthy controls exploring the relationship between the excitability of the motor cortex assessed by transcranial magnetic stimulation (TMS) and brain morphology evaluated by voxel based morphometry (VBM).

Excitability was evaluated using paired TMS to elicit a short-latency intracortical inhibition (SICI) in the motor cortex of the hand muscles to assess the acute effects of the GPi DBS^18,19^. This technique was previously used in dystonia patients to show affected cortical excitability in various dystonic syndromes^20,21^ however no acute effects of GPi DBS on the SICI were detected^22–24^. On the other hand, the effects of long-term exposure of the GPi DBS on brain morphology are unknown. We expected that GPi DBS may induce local grey matter density changes especially in areas belonging to the sensorimotor circuits.

Instead of focusing on a specific type of dystonia we purposefully recruited a heterogeneous group of patients with cervical or generalized dystonia regardless of etiology, expecting a common mechanism of GPi DBS in all dystonia patients. We stratified our patients according to the clinical benefit of chronic treatment obtained by GPi DBS in comparison to their preoperative state into three groups – non-responders, partial responders and good responders. We hypothesized that the pattern of motor cortex excitability and brain morphology in patients with a poor clinical response to GPi DBS will differ from patients with a partial or good response which should be close to findings in a healthy group.

## Results

Twenty two patients participated in the TMS part of the study and in 19 of them a T1-weighted brain MRI was obtained. Their electrophysiology and brain morphology parameters were then compared with data from 22 matched healthy controls.

The long-term clinical benefit of GPi DBS treatment expressed as a relative change in the adjusted dystonic score (derived from the Burke-Fahn-Marsden Dystonia Rating Scale – BFMDS motor score or from the Toronto Western Spasmodic Torticollis Rating Scale – TWSTRS severity score) between the GPi DBS ON condition and the preoperative state was 38.5±31.0% (p < 0.001). Eight patients with a clinical improvement >50% were considered as good responders (GR), five patients with an improvement of 25–50% as partial responders (PR) and nine patients with improvement <25% as non-responders (NR). Disruption of the GPi DBS for two hours caused 18.4±20.3% worsening (p < 0.001) of the adjusted dystonic score.

There was no correlation between the relative preoperative/postoperative change of the adjusted dystonic score in the GPi DBS ON state and the duration of chronic GPi DBS stimulation (p = 0.73) nor any correlation between the GPi DBS ON/OFF relative change of the adjusted dystonic score assessed postoperatively and the duration of chronic GPi DBS stimulation (p = 0.41).

### Motor thresholds

The resting and active motor threshold did not differ between control subjects and patients for any of the factors *‘DBS state’* or clinical *‘benefit’*.

### SICI

The linear mixed effects regression model of the SICI (expressed as a percentage of amplitude of MEP elicited by a single unconditioned TMS pulse) was significant for the factor *‘DBS state’* (F = 11.43, p < 0.001), *‘intensity’* of the conditioning TMS stimulus related to the active motor threshold (AMT) (F = 7.58, p < 0.001), and *‘benefit’* (F = 5.42, p < 0.05) with significant interactions for *‘state* x *benefit’* (F = 12.19, p < 0.001) and interaction *‘DBS state* x *intensity’* (F = 3.03, p < 0.05). As the factor *‘muscle’* was not significant, the SICI from the APB and ADM muscles was considered together.

**Figure 1:**
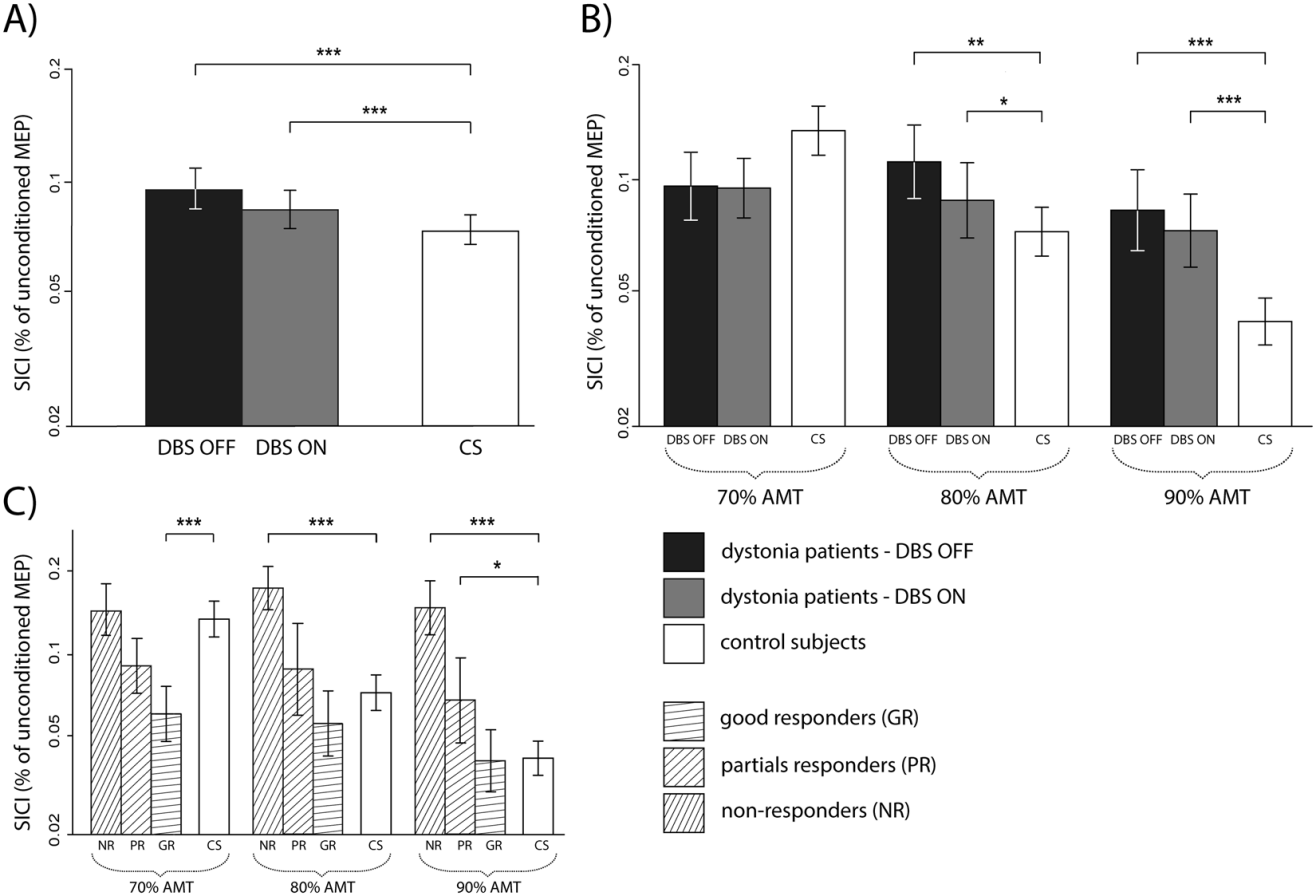
Short-latency intracortical inhibition (SICI) in dystonic patients in both states (GPi DBS ON and OFF) and in control subjects. The SICI was expressed as % amplitude of MEP (±SEM) elicited by a single unconditioned TMS pulse. (**A**) Dystonic patients in both states (GPi DBS OFF and ON) have less effective SICI (=higher % of unconditioned MEP) than controls with no difference between DBS ON and OFF states. The SICI was averaged from both the APB and ADM muscles and from all three AMT intensities (70%, 80%, 90%) of the conditioning stimulus in the paired TMS paradigm. (**B**) The SICI was more effective with an increasing AMT intensity of the conditioning TMS stimulus in control subjects. Dystonic patients have less effective SICI (=higher % of unconditioned MEP) with 80% and 90% of the AMT intensity of the conditioning stimulus in both GPi DBS states. The SICI was averaged from both the APB and ADM muscles. (**C**) Non-responders and partial responders had less effective SICI (=higher % of unconditioned MEP) with 90% AMT intensity of the conditioning stimulus in comparison with control subjects. Good responders had more effective SICI (=lower % of unconditioned MEP) with 70% AMT intensity of the conditioning stimulus. The SICI was averaged from both the APB and ADM muscles and from both the GPi DBS OFF and ON states. APB = Abductor Pollicis Brevis muscle, ADM = Abductor Digiti Minimi muscle, AMT = Active Motor Threshold, GR = good responders (>50% benefit), PR = partial responders (25–50% benefit), NR = non-responders (<25% benefit), CS = control subjects, *p < 0.05, **p < 0.01, ***p < 0.001.

Subsequent *post hoc* tests revealed that dystonia patients had less efficient SICI (=higher % of unconditioned MEP) than control subjects (F = 4.93, p < 0.001) with no significant difference between the GPi DBS ON and OFF. The contrasts between control subjects and dystonia patients were significant for both the GPi DBS OFF state (F = 7.49, p < 0.001) as well as the GPi DBS ON state (F = 7.00, p < 0.001) (Fig. 1A). Further exploration of the *‘DBS state* x *benefit’* interaction showed that the SICI gradually decreased (=% of unconditioned MEP gradually increased) from good responders, to partial responders and to non-responders. Good responders had more efficient SICI (=lower % of unconditioned MEP) than control subjects (F = 3.25, p < 0.01), whereas non-responders had less efficient SICI (=higher % of unconditioned MEP) than control subjects (F = 5.83, p < 0.001).

When considering the interaction of *‘DBS state* x *intensity’*, the *post hoc* tests showed that dystonia patients had less effective SICI (=higher % of unconditioned MEP) than control subjects only with the 80–90% AMT intensity of the conditioning stimulus in both the GPi DBS OFF state (80% AMT intensity: z = 3.596, p < 0.01; 90% AMT intensity: z = 4.511, p < 0.001) and the GPi DBS ON state (80% AMT intensity: z = 3.197, p < 0.05; 90% AMT intensity: z = 4.486, p < 0.001) whereas no differences for the 70% AMT intensity were found (Fig. 1B).

*Post hoc* exploration of the interaction of *‘benefit* x *intensity’* revealed that with 80% and 90% of the AMT intensity of the conditioning stimulus, SICI did not differ in good responders from control subjects, but with 70% of the AMT intensity the SICI was significantly more effective in good responders than in controls (F = 8.73, p < 0.001) (Fig. 1C).

### MEP onset latency

The MEP onset latency was evaluated with the linear mixed effects regression model. The significant factors were *‘DBS state’* (F = 15.86, p < 0.001), *‘benefit’* (F = 14.05, p < 0.01) and *‘muscle’* (F = 11.57, p < 0.001) with the only significant interaction *‘DBS state* x *benefit’* (F = 30.22, p < 0.001). The intensity of the conditioning TMS stimulus had no effect on the MEP onset latency.

**Figure 2:**
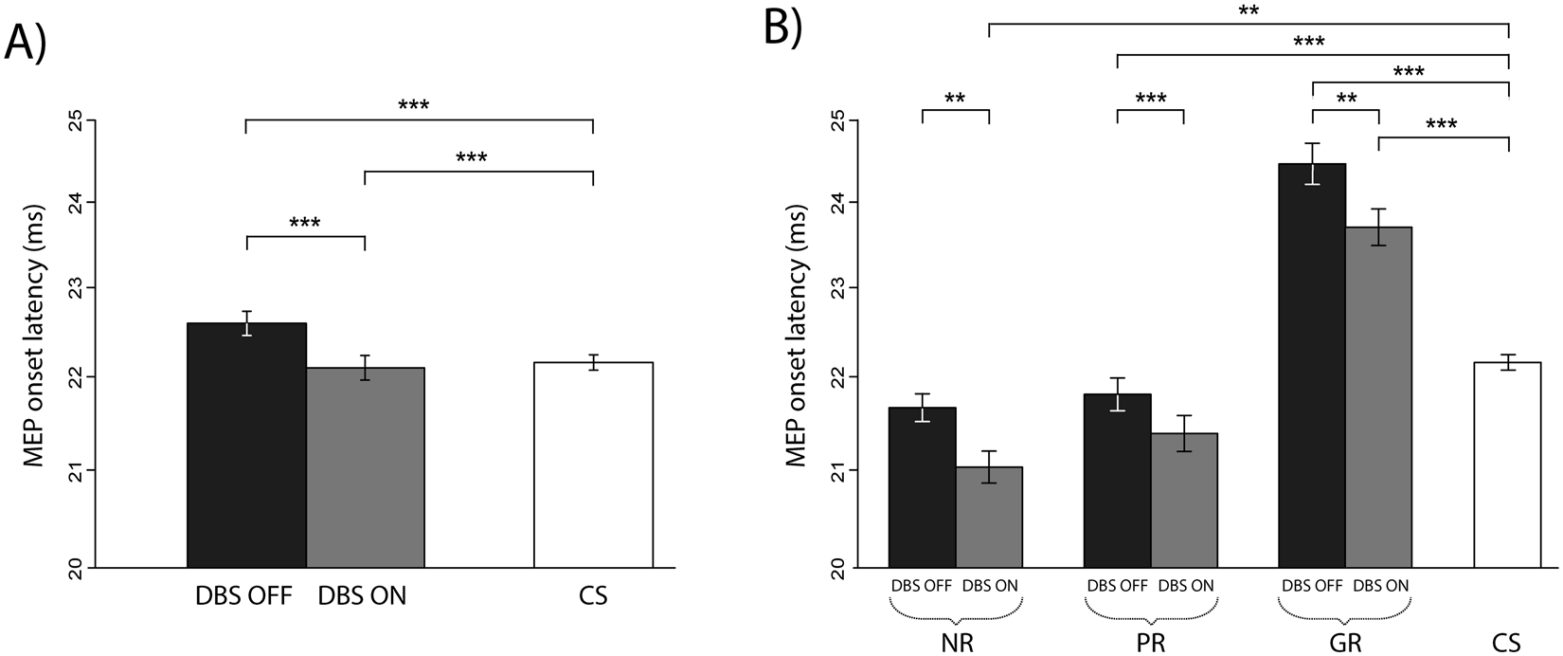
MEP onset latency in dystonic patients during both states (GPi DBS ON and OFF) in comparison with control subjects. The MEP onset latency was expressed as an average (±SEM) from both the single and paired pulse TMS paradigms and from both the APB and ADM muscles. (**A**) Control subjects had shorter MEP onset latency than dystonic patients in both GPi DBS states. Switching the GPi DBS to ON caused a shortening of the MEP onset latency in comparison with the OFF state. (**B**) The MEP onset latency differed according to the clinical benefit of the GPi DBS treatment in comparison with the preoperative state. While good responders had the MEP onset latency longer than control subjects, partial responders and non-responders had the MEP onset latency shorter than control subjects. APB = Abductor Pollicis Brevis muscle, ADM = Abductor Digiti Minimi muscle, GR = good responders (>50% benefit), PR = partial responders (25–50% benefit), NR = non-responders (<25% benefit), CS = control subjects, **p < 0.01, ***p < 0.001.

The *post hoc* exploration of the factor *‘DBS state’* showed that dystonia patients in comparison with control subjects had longer MEP onset latency in both GPi DBS states (OFF: F = 32.67, p < 0.001; ON: F = 22.66, p < 0.001)(Fig. 2A). The MEP onset latency in dystonia was on average 0.57±(SD)1.21 ms shorter in the GPi DBS ON compared to the OFF state from both the APB and ADM muscles and both the single and paired TMS pulses (F = 16.72, p < 0.001).

This GPi DBS-related shortening of the MEP onset latency was observed in good responders (z = −3.70, p < 0.01), partial responders (z = −5.78, p < 0.001) and even in non-responders (z = −3.89, p < 0.01). The longest MEP onset latency was found in good responders, shorter in partial responders and the shortest in non-responders (Fig. 2B). The good responders had a longer MEP onset latency than control subjects in both GPi DBS states (OFF: z = 8.06, p < 0.001; ON: z = 4.55, p < 0.001).

### Spontaneous muscle activity

Pre-activation activity of the APB and ADM muscles during the paired pulse TMS protocol in two of the analysed intervals did not change between the GPi DBS states. The number of sweeps exceeding the individually calculated threshold did not change for neither of the 10-ms interval (i) preceding the conditioning TMS pulse (APB OFF vs. ON: 3.4 vs. 6.3, p = 0.175, ADM OFF vs. ON: 6.0 vs. 11.5, p = 0.060) nor for the 20-ms interval (ii) following 80 ms the conditioning TMS pulse (APB OFF vs. ON: 6.5 vs 9.7, p = 0.265, ADM OFF vs. ON: 9.5 vs. 13.7, p = 0.094). Accordingly, the area under curve of the rectified EMG in both of the analysed intervals did not change between the GPi DBS states (interval i: p = 0.275, interval ii: p = 0.339).

### VBM

**Figure 3:**
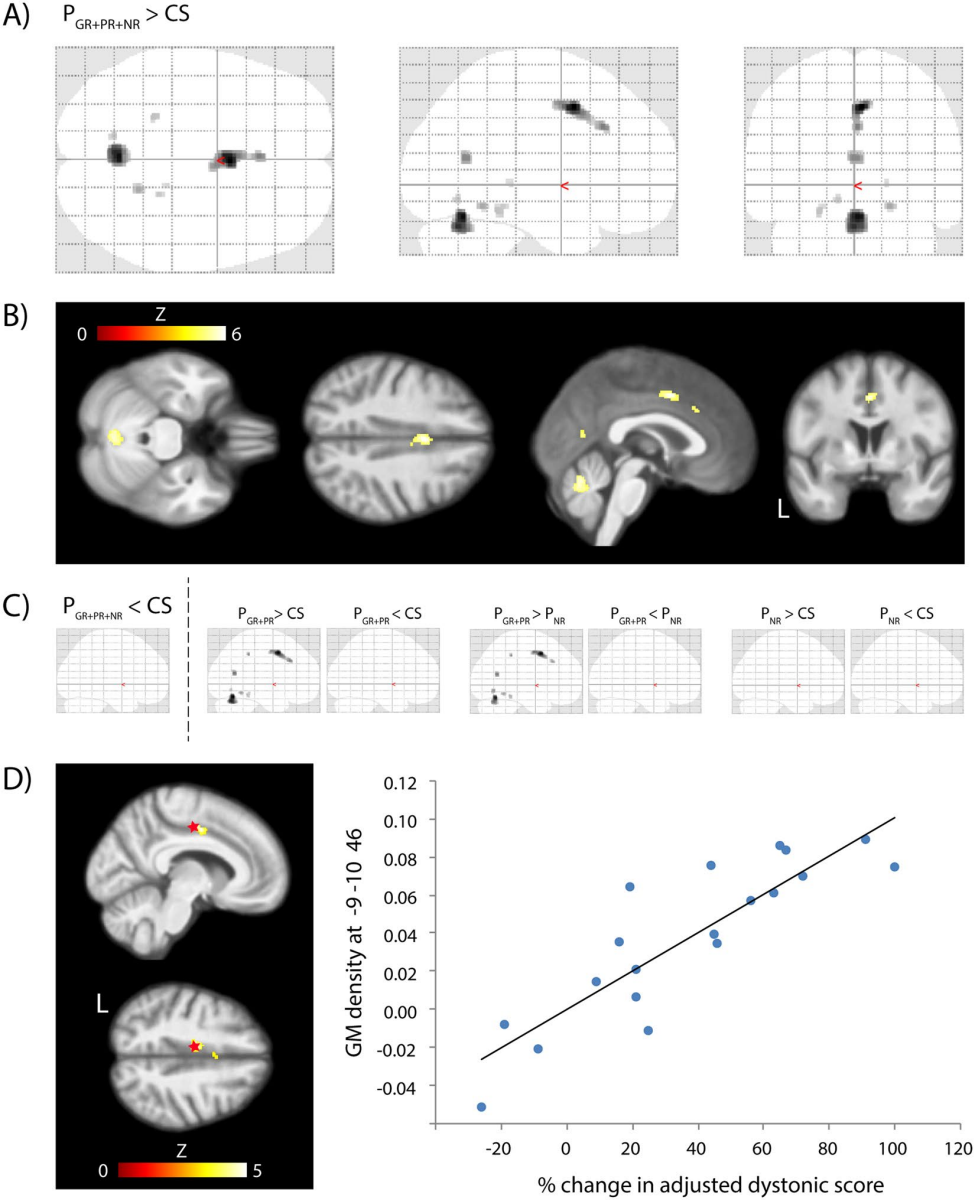
Comparison of GM density between GPi DBS treated dystonia patients and control subjects. Age and total intracranial volume were used as nuisance vectors. Glass brain (**A**) and perpendicular projections (**B**) on an average T1-weighted brain MRI showing higher GM density in the cluster involving the SMA and anterior/middle cingulate and in the vermis of the cerebellum in all patients (P_GR+PR+NR_) compared to CS (p < 0.05 FWE corrected at peak level). (**C**) Clusters similar to the P_GR+PR+NR_ > CS contrast were also obtained in contrasts P_GR+PR_ > CS and P_GR+PR_ > P_N_ suggesting that the GM was selectively increased in good responders (GR) and partial responders (PR) compared to non-responders (NR) or control subjects (CS)(p < 0.05 FWE corrected at peak level). (**D**) – Correlation of the GM with % change in the adjusted dystonic score at the voxel x = -9, y = 10, z = 46 (red star) was significant (r = 0.87, p < 0.000001) in the middle cingulate cortex and ventral SMA where the increase of GM density with an increasing change of the adjusted dystonic score was found (image p < 0.05 FWE corrected at cluster level).

Dystonia patients in comparison with control subjects showed an increased GM density in a cluster involving the pre-supplementary motor area (pre-SMA) and middle cingulate gyrus, and, additionally, in the cerebellar vermis (Fig. 3A,B, Supplementary Table 1). An almost identical GM density increase was also detected in responders (good and partial) when compared to non-responders or to control subjects (Fig. 3C). The correlation of the GM density with the relative change of the adjusted dystonic score in patients with chronic GPi DBS in comparison with the condition before implantation showed a similar cluster involving the middle cingulate gyrus and ventral SMA (Fig. 3D).

**Figure 4:**
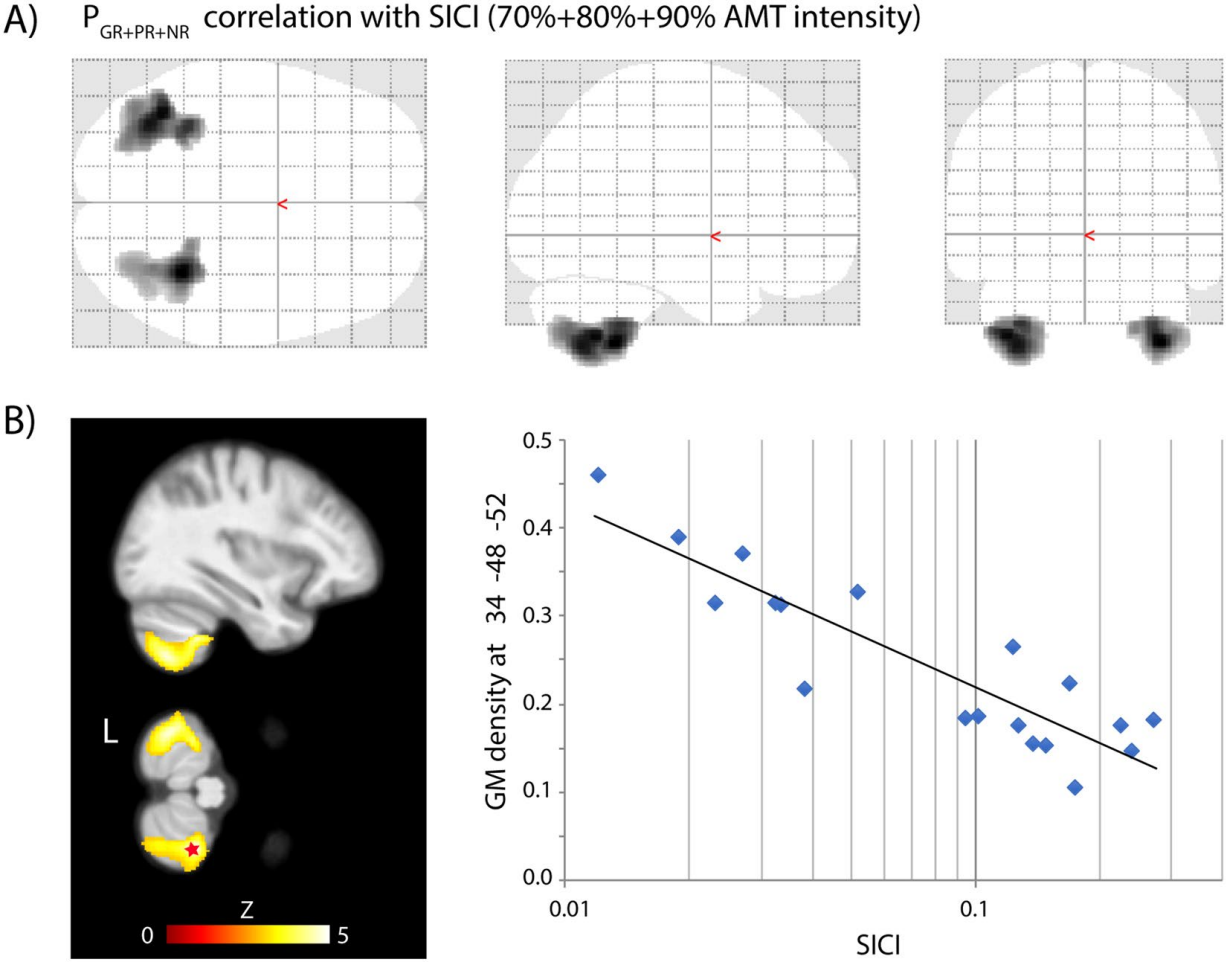
Inverse correlation of the SICI with GM density in GPi DBS treated dystonia patients. Age and total intracranial volume were used as nuisance vectors. The average SICI (elicited with 70%, 80%, 90% AMT intensity of the conditioning stimulus in the paired TMS paradigm in both the APB and ADM muscles and in both the GPi DBS OFF and ON states) was used in the model. (**A**) Glass brain and two projections (**B**) on an average T1-weighted brain MRI show the clusters in the cerebellar hemispheres (p < 0.05 FWE corrected at cluster level) whose GM density inversely correlated with the SICI. The linear regression at the voxel of maximum correlation x = 34, y = −48, z = −52 is shown (r = −0.77, p < 0001). (**C**) Three separate results of *post hoc* analyses showing the contrast between GM in good responders (GR) and control subjects (CS) according to various AMT intensity (70%, 80% and 90%) of the conditioning stimulus (p < 0.001). The GM contrast was maximally pronounced with 70% intensity, less with 80% intensity and no difference was detected with 90% intensity. These gradual differences in GM density are similar to contrasts of SICI between good responders (GR) and control subjects (CS).

Analysis of the SICI and GM density showed in dystonia patients a significant relationship symmetrically in the ventral portions of both cerebellar hemispheres (VIIb, VIII, IX, crus II) in which GM density inversely correlated with the mean SICI regardless of the conditioning stimulus intensity, muscle tested or DBS state (Fig. 4A,B, Supplementary Table 2). Conversely, no correlation between the SICI and GM density was found in control subjects.

**Figure 5:**
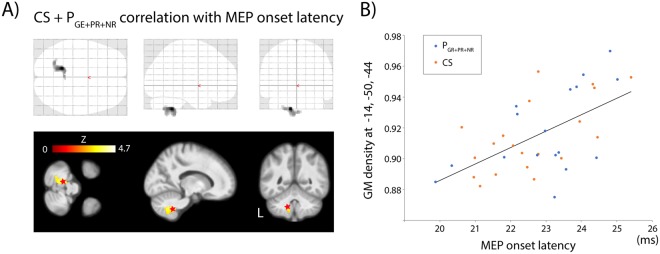
Correlation of the MEP onset latency with the GM density in both groups of subjects. The MEP was elicited by the single TMS pulse in the APB muscle in control subjects and in GPi DBS treated patients during OFF state. (**A**) Glass brain and three projections on average T1-weighted brain MRI show the cluster of positive correlation in the left cerebellum (9, crus 1) (p < 0.05 FWE corrected for volume of cerebellum). (**B**) Regression line between the MEP onset latency and the GM density at voxel with maximum correlation [−14, −50, −44](r = 0.58, p < 0.001).

Analysis of the MEP onset latency and GM density showed negative results in both groups of subjects. However, when a less conservative approach was applied, i.e. when the volume correction was limited to the cerebellum (a region in which previous analyses revealed strong findings), results became significant. When combining data from both groups of subjects together, a positive correlation between GM density and the mean MEP onset latency was found in the cluster near the cerebellar vermis with this procedure (Fig. 5) (Supplementary Table 3).

## Discussion

Our dystonia patients treated with chronic GPi DBS showed distinct changes in the short latency cortical inhibition (SICI) as well as in the MEP onset latency accompanied by significant brain morphology changes. In general, the SICI was less effective in patients in comparison with control subjects. This phenomenon, interpreted also as an increase in the excitability of the primary motor cortex was seen in all patients, despite the heterogeneity of dystonic syndromes, and it was independent of the GPi DBS state (Fig. 1A). Moreover, the SICI in dystonia patients had a close relationship with brain morphology, showing an increasing GM volume of cerebellar hemispheres in association with the decreasing amplitude of the MEP elicited by paired TMS (Fig. 4). This corroborates a crucial role of the cerebello-thalamo-cortical projections in control of the excitability of the motor cortex.

In addition to chronic effects, we observed the acute effects of GPi DBS manifested as a shortening of the MEP onset latency with both single and paired TMS protocols detected in all dystonia patients (Fig. 2). Surprisingly, the patients with good clinical benefits were characterized by longer MEP onset latency in both GPi DBS states than other patients and controls, which was presumably related to more complex rebuilding of the motor cortex. We also took in account the possibility that dystonia patients can react differently when switching the stimulation off and on depending on the duration of chronic DBS. The reason for this variability is probably related to the individual level of synaptic plasticity in each patient. However, we did not find any correlation between the clinical benefit of the GPi DBS and the GPi DBS ON/OFF and the duration of chronic GPi DBS stimulation.

Our dystonia patients showed impaired SICI of the motor cortex in comparison to control subjects with no clear impact of a short-term disruption of the GPi DBS (Fig. 1A). The SICI is probably generated by synaptic inhibitory mechanisms mediated by interneurons in the primary motor cortex involving gamma-aminobutyric acid A receptors^18,25^. The increased cortical excitability assessed by the SICI has been previously reported in patients with focal^20,26^, as well as with generalized dystonia^23,27^. The SICI phenomenon is usually attributed to dystonia overflow or to impaired ability to suppress unwanted movements at the cortical level^20^. Our observation of low SICI may then reflect the general abnormality of the motor cortex in dystonia and not the direct influence of GPi DBS. Indeed, the SICI gradually improved in dystonia patients with the GPi DBS to normal values during a period of several months after its initiation^28^ whereas a sudden switch off for a couple of hours^22^, for two days^23^ or even for several weeks^24^ left the SICI unaffected.

The magnitude of SICI depends critically on the intensity of both the conditioning and testing stimulus^25^. When the intensity of the conditioning stimulus is related to AMT, maximum SICI usually occurs at intensities of 90–100% AMT and decreases with the lowering intensity of the conditioning stimulus^29^. This was observed in our control subjects but not in dystonia patients (Fig. 1B). They reached the same level of SICI as healthy controls only with a lower intensity (70%) of the conditioning stimulus, whereas their ability of suppression at a higher intensity (80–90%) was significantly impaired (Fig. 1B).

The different behavior of the SICI was further explained by a stratification of patients according to the clinical benefit of the GPi DBS treatment measured with respect to the preoperative state considering the long-term effects of GPi DBS (Fig. 1C). The impaired SICI elicited with higher intensities of the conditioning stimulus was observed especially in non-responders whose clinical improvement did not exceed 25% of their preoperative dystonic score. On the other hand, good responders whose clinical benefit was higher than 50%, had a similar cortical excitability to healthy controls only when higher intensities of the conditioning pulse were used, but they overreacted to conditioning stimulus of lower intensity causing a stronger SICI than in control subjects. Cortical excitability of partial responders was somewhere in the middle between good responders and non-responders showing insufficient SICI only for the highest intensity of the conditioning stimulus (Fig. 1C). All this suggests fundamental differences in motor cortex excitability according to the clinical response to GPi DBS treatment showing a very high sensitivity of the motor cortex to conditioning stimuli in good responders and poor reactivity in non-responders. Our results suggest that only good responders are well enough equipped to effectively suppress stimuli coming from other regions to the motor cortex, which would otherwise be converted to dystonic movements. As this study was originally designed as cross-sectional, we can hardly decide whether good responders underwent a ‘hardwire’ reorganization due to chronic GPi DBS or if their motor cortex already had this capability before implantation.

Variable GM volume changes of the SMA^16,30^ or cerebellum^17,31^ were previously described in patients with focal dystonia, but our study is the first one reporting volume changes in dystonia patients treated by DBS. Our VBM analysis showed that the long-term clinical benefit of GPi DBS (Fig. 3) and the level of motor cortex excitability assessed by SICI (Fig. 4) were reflected in the structural changes of regions belonging to the motor network. Good and partial responders showed a higher local GM volume in the vermis of the cerebellum than control subjects and non-responders. These responders also had a GM hypertrophy in the ventral SMA and middle cingulate cortex, in which the GM volume positively correlated with postoperative clinical improvement. Our patients who had better antidystonic effects of the GPi DBS, obviously had a larger amount of GM tissue in the SMA. It seems that brain of dystonia patients treated by the GPi DBS share common hallmarks expressed in local cortical volume despites variability of underlying pathology. On the other hand, the various etiology is the reason why brain morphology differs in another aspects among dystonia patients. For example, generalized dystonia patients due to pantothenate kinase-associated neurodegeneration (PKAN) were described as having decreased GM volume in the basal ganglia and SMA^32^. Symptomatic DYT1 mutation carriers and non-DYT1 adult patients show differences in volume of the putamen^16^ suggesting specific remodeling of the motor network according to dystonia origin.

Our observations are in agreement with previous literature showing the critical role of the SMA^33,34^ and cerebellum^35,36^ in the pathophysiology of dystonia. The SMA is involved in motor learning and cognitive control of movement^37^ or perhaps even in the suppression of unwanted motor acts^38^. In animal models of dystonia, the SMA showed an increased excitability^39,40^ and abnormal proprioceptive fields^33^. Functional imaging revealed that the SMA in focal dystonia is usually hypoactive^41^, whereas activation arises as a consequence of successful treatment^42^. Our results further contribute to this field suggesting that the SMA volume is quantitatively associated with the suppression of involuntary movements corroborating compensatory the role of the SMA in dystonia patients treated by GPi DBS.

Different regions of the cerebellum were related to clinical improvement and the SICI phenomenon. While the hypertrophy of the upper vermis was detected in dystonia patients with a good or partial response to GPi DBS (Fig. 3A–C), the variations of SICI were related to the volume of cerebellar hemispheres (Fig. 4). As the vermis belongs to the motor part of the cerebellum^43^, we may assume that the positive outcome of chronic GPi DBS was associated with a more efficient engagement of the cerebellum via dentate-thalamo-cortical projection^44^. Decreased integrity of this pathway assessed by probabilistic tractography was already described in relationship with a higher penetrance of dystonia^45^. Activation in this pathway may also explain some of the inhibitory and excitatory mechanisms in the human motor cortex related to the TMS of the cerebellum in healthy subjects^46,47^. However, these effects are variable according to the TMS technique^46,48,49^.

Based on these reports proving the distant influence of the cerebellum on the excitability of the motor cortex it is not surprising that we found a relationship between morphology changes in the cerebellum and the SICI of the motor cortex. From all GM brain regions, only the inferior portion of both cerebellar hemispheres showed an inverse correlation between volume and SICI (Fig. 4). In dystonia patients, the SICI was more effective with a larger volume of the cerebellar lobule VIII, which is well connected to the motor cortex and with a larger volume of the cerebellar crus II and lobule VIIb, which are regions with connectivity to the prefrontal cortex and posterior parietal cortex^50,51^. The inhibitory function of the cerebellum may thus influence the excitability of the motor cortex directly via thalamus or indirectly through multiple connections from adjacent prefrontal or parietal cortices.

Dystonia patients with the GPi DBS ON showed a decreased MEP onset latency compared to the OFF state (Fig. 2A) suggesting an immediate change in the functional activity of the motor cortex associated with the acceleration of cortical processing and facilitation of the motor response. As the observed changes appeared not only with paired-TMS but also with the single pulse paradigm we may assume that the acceleration of the motor response with acute GPi DBS differed from the mechanism of improved SICI detected in good responders with chronic GPi DBS. In contrast to SICI, the MEP onset latency shortening appeared in each group of patients (Fig. 2B), supporting a more universal underlying effect unrelated to the clinical outcome of GPi DBS.

The observed shortening of the MEP onset latency could potentially be a consequence of muscle pre-activation by underlying dystonic activity of the hand muscles especially in patients with generalized dystonia, which may decrease with the GPi DBS ON. Accordingly, the voluntary pre-contraction may facilitate the MEP response accompanied with its earlier onset^52^. We believe, that neither of those mechanisms were important confounding factors in our study as the spontaneous muscle activity analysed in the two intervals did not change between the GPi DBS states.

The shortening of the MEP onset latency has already been seen in patients with Parkinson’s disease treated by DBS of the subthalamic nucleus shortly after surgery with the external stimulator switched off in comparison with the preoperative state^53^ as well as in dystonia patients treated with GPi DBS in comparison with healthy controls^22^. However, the shortening in the latter study was present regardless of the GPi DBS state suggesting a methodological artifact caused by TMS-induced currents in the implanted electrode or in connection leads beneath the coil^54^. To suppress this artifact in our study, the TMS-coil was purposefully positioned far from the connection leads. Nevertheless, TMS-induced currents in the electrodes can hardly explain the observed difference in the MEP onset latency between the GPi DBS ON and OFF states as the induced currents should not change between DBS states. The reason why the previous study^22^ did not detect a difference in the MEP onset latency might be related to a lower number of patients or to an insufficiently long disruption of the GPi DBS.

The beginning of the MEP depends on the spatial organization of the neuronal network with respect to the direction of currents induced by the TMS-coil selectively activating a certain population of interneurons and pyramidal cells^55^. As we used only one orientation of the coil we cannot reliably distinguish which neuronal population of the motor cortex was preferentially stimulated. However, with the position of the coil in the posterior to anterior direction, cortical interneurons are more likely to be activated than with the coil positioned in medial-lateral direction^25,56^. The reason for the acute GPi DBS-related shortening of the MEP onset latency in our study may then rely on pre-excitation of these cortical interneurons as a consequence of modulatory input projecting from the GPi via thalamus^57^.

However, there could be another explanation suggesting two different motor networks with different processing times that can be engaged in each of the GPi DBS states. This hypothesis is based on the assumption that the processing time within the motor cortex depends on the complexity of the neuronal network. A poor network with a lower number of interneurons (or synapses) should process action faster than a complex network containing a larger number of interneurons because of multiple synaptic delay. In addition, a complex network should be associated with a larger GM volume than a poor network. In accordance with this hypothesis, we observed that the volume of the medial portion of the cerebellum (Crus I, lobule IX, Fig. 5A) gradually increased with the MEP onset latency (Fig. 5B). Indeed, the crus I is connected predominantly with the prefrontal as well as with the contralateral motor cortex^50,51^ further emphasizing the inhibitory role of the cerebello-thalamo-cortical projections.

We believe that a higher complexity of the neuronal network is responsible for differences in the MEP onset latency among patients with respect to the clinical outcome of the GPi DBS. We showed that the shortest MEP onset latency was observed in non-responders, followed by partial responders and good responders (Fig. 2B). Non-responders may lack “good” plasticity to rebuild the motor cortex in response to chronic GPi DBS. Their motor network was poor resulting in a fast processing time and in the shortest MEP latency. On the other hand, good responders were able to induce plastic changes creating a more complex network with multiple synapses allowing better control of dystonic movement but associated with a significant prolongation of cortical processing time, which was even longer than in control subjects.

Our hypothesis of different complexity networks fits with previous papers suggesting that the MEP onset latency may serve as a biomarker of plasticity^58,59^. They showed that the after-effect of continuous TMS theta burst stimulation delivered to the motor cortex depends on the MEP onset latency recorded before. Healthy subjects with longer MEP onset latency reacted with better inhibition to this protocol, which resembles our results in dystonia patients. Our patients with longer MEP onset latency were those with better intracortical inhibition and with better clinical benefits.

There were several limitations in the study. The cross-sectional design with only actual electrophysiology and imaging parameters cannot distinguish between preoperative conditions and induced plastic changes due to chronic GPi DBS. The heterogeneity of the patients with variable concomitant therapy was advantageous for searching for common mechanisms of GPi DBS but disadvantageous for detection of changes specific for a particular dystonic syndrome. On the other hand, we cannot exclude that some aspects of GPi DBS may differ among dystonic syndromes of various etiology and that abnormalities in brain morphology specific to dystonic syndromes like PKAN or post-anoxic encephalopathy may compromise the VBM results. Better detection of ongoing background muscle activity with potentially underlying preexcitation of the motor cortex due to voluntary and dystonic activity is needed in future studies, as the short detection intervals used in our study may not be sufficiently sensitive. And finally, two hours disruption of GPi DBS is not long enough to avoid the after-effect of GPi DBS potentially lowering the chances for detecting a contrast between both stimulation states. Despites previous research using short-lasting switching paradigms^22^, long-term plasticity changes induced by GPi DBS could not be easily detected, as two hours of disruption may have different pathophysiological meaning in patients with short and long durations of chronic GPi DBS therapy. Nevertheless, this was probably not the case of our study, as the change in the adjusted clinical score between the GPi DBS OFF and ON states nor the change between preoperative and postoperative clinical involvement were unrelated to the time elapsed from the implantation.

## Conclusions

Our study showed a close relationship between cortical excitability of the motor cortex, grey matter volume and the clinical benefit of the GPi DBS in various dystonia syndromes. While its acute effect was associated with shortening of the motor response regardless of clinical efficacy, patients with good clinical response to chronic GPi DBS had more effective intracortical inhibition than partial responders or non-responders, reaching its level like in healthy subjects. This ability to inhibit the abnormally sensitive motor cortex was quantitatively associated with growing volume of the cerebellar hemispheres supporting participation of cerebello-thalamo-cortical projection which together with mesial increase of grey matter volume may contribute to better anti-dystonic effects of pallidal stimulation. It remains to be clarified, whether these excitability and morphometry parameters may serve as a biomarker of future clinical response to GPi DBS in dystonia or if their changes were induced as a consequence of this treatment.

## Methods

We included 22 patients (13 F, 9 M, aged 51±(SD)17 years) with dystonia of various body distribution (15 generalized, 7 cervical) and etiology (14 idiopathic, 2 PKAN, 2 DYT-1, 2 post-anoxic, 1 PINK1, 1 KMT2B mutation) with a disease duration of 16±(SD)6 years. For comparison, we included 22 age- and gender-matched healthy controls (13 F, 9 M, aged 51±(SD)17 years) with no history of neurological or psychiatric disorders. All patients were treated by chronic bilateral GPi DBS and were examined 1–9 years (or 4±(SD)2 years) after the implantation of the electrodes (Table 1). All subjects gave their written informed consent to participate and the study was approved by the local ethics committee of the General Faculty Hospital in Prague in compliance with the Declaration of Helsinki.

**Table 1:** Descriptive data of the dystonia patients.

Age at Onset (years)	Age at Surgery (years)	Etiology	Body Distribution	Dystonia Duration (years)	GPi DBS Duration (years)	BFMDS preop	BFMDS GPi DBS ON	BFMDS GPi DBS OFF	Medication (daily dose)
adulthood	56	idiopathic	generalized	12	4	39	28.5	28.5	biperiden 16 mg,
childhood	20	DYT 1	generalized	25	6	27	2.5	4.5	0
adulthood	51	PINK 1	generalized	28	3	50	22.5	32	escitalopram 10 mg, Botulinum toxin A
childhood	26	post-anoxic	generalized	16	1	47.5	46	51	clonazepam 2.5 mg, baclofen 20 mg, biperiden 3 mg, valproate 600 mg, venlafaxine 150 mg, Botulinum toxin A
childhood	13	DYT 1	generalized	11	5	28.5	14	21	0
childhood	18	PKAN	generalized	18	9	77.5	55	61	clonazepam 1.5 mg, biperiden 3 mg, pantothenate 500 mg, Botulinum toxin A
childhood	16	PKAN	generalized	16	8	70.5	80	82	biperiden 18 mg, clonazepam 3.75 mg, baclofen 30 mg, panthotenate 500 mg, citalopram 40 mg, Botulinum toxin A
infancy	30	post-anoxic	generalized	32	3	50.5	47.5	47.5	0
adulthood	65	idiopathic	generalized	11	4	15	x	12	mirtazapine 30 mg
adulthood	69	idiopathic	generalized	13	3	8	0	0	0
adulthood	53	KMT2B	generalized	15	5	20.5	7	x	0
adulthood	36	idiopathic	generalized	18	6	29	24.5	24.5	clonazepam 1 mg, amitriptyline 50 mg, citalopram 40 mg, gabapentin 1800 mg
adulthood	52	idiopathic	generalized	11	2	33	13.5	15.5	trazodone 150 mg, venlafaxine 150 mg,
adulthood	61	idiopathic	generalized	11	3	21	12.5	15.5	bromazepam 2.25 mg, venlafaxine 150 mg,
adulthood	73	idiopathic	generalized	8	1	16	15.5	18.5	levodopa 1250 mg, carbidopa 125 mg, clonazepam 0.75 mg, sertraline 100 mg
						**TWSTRS preop**	**TWSTRS GPi DBS ON**	**TWSTRS GPi DBS OFF**	
adulthood	41	idiopathic	cervical	23	4	24	17	19	alprazolam 0.5 mg, citalopram 60 mg, Botulinum toxin A
adulthood	48	idiopathic	cervical	18	4	20	16	19	baclofen 30 mg, venlafaxine 150 mg, promethazin 25 mg,
adulthood	47	idiopathic	cervical	10	1	26	22	22	clonazepam 1 mg, primidone 500 mg, citalopram 20 mg, Botulinum toxin A
adulthood	38	idiopathic	cervical	12	5	24	5	19	0
adulthood	74	idiopathic	cervical	13	3	25	22	22	clonazepam 0.25 mg, citalopram 10 mg, Botulinum toxin A
childhood	52	idiopathic	cervical	11	4	29	6	13	0
adulthood	51	idiopathic	cervical	16	7	24	14	22	biperiden 2 mg, clonazepam 0.5 mg, sertraline 100 mg,

### Experimental design

The study was designed as an open-label with two un-blinded sessions performed on the same day. The session was started either: (i) at least two hours after switching the GPi DBS ON or (ii) at least two hours after switching the GPi DBS OFF in a random order. The clinical assessment and electrophysiological testing were performed in each session - except for two patients where testing was performed in only one condition. Patient #11 with generalized dystonia was not tested after switching the stimulation off because of acute worsening of dystonia. Patient #9 refused to continue with the stimulator on state due to subjective intolerance of the study procedure. Both of these patients were kept in the patient’s cohort as their exclusion did not affect the significance of the results. Healthy controls were assessed in only one session. Brain MRI was obtained a different day for all subjects with the exception of three patients, which were excluded from the VBM part of the study: patients #1 and #11 because of severe motion artifacts on the T1-weighted brain MRI and patient #9 in whom the MRI was not performed because of lack of a follow up.

### GPi DBS specification

All patients were implanted bilaterally with a quadripolar electrode to the posteroventrolateral portion of the GPi according to a previously described procedure^60^ using the anatomical target: 17–21 mm laterally from the midline, 2–3 mm anteriorly from the mid-commissural point and 4–5 mm beyond the AC-PC line just above the individual’s optic tract. In the GPi DBS ON state, patients were examined using their optimal DBS parameters (Table 2).

**Table 2:** The GPi DBS parameters.

Patient	Right GPi DBS			Left GPi DBS			Implantable Generator	Electrodes
	Amplitude	Pulse Width [µs]	Frequency [Hz]	Amplitude	Pulse Width [µs]	Frequency [Hz]		
1	2.6 mA	400	70	1.9 mA	400	70	Brio, St.Jude	6148
2	1.5 V	450	100	1.5 V	450	100	RC Activa, Medtronic	3389
3	1.65 mA	212	130	1.35 mA	212	130	Brio, St.Jude	6148
4	0.9 mA	162	50	1.2 mA	162	50	Brio, St.Jude	6148
5	2.0 V	240	130	2.6 V	240	130	RC Activa, Medtronic	3389
6	1.8 V	450	130	1.8 V	450	130	RC Activa, Medtronic	3389
7	1.4 V	450	130	1.3 V	450	130	RC Activa, Medtronic	3389
8	1.6 mA	170	90	1.9 mA	180	90	RC Activa, Medtronic	3389
9	1.0 V	450	200	1.0 V	450	200	RC Activa, Medtronic	3389
10	2.5 V	180	130	2.5 V	180	130	Kinetra, Medtronic	3389
11	1.8 V	210	130	1.8 V	210	130	RC Activa, Medtronic	3389
12	2.4 V	450	130	2.4 V	450	130	RC Activa, Medtronic	3389
13	3.0 mA	300	130	3.0 mA	300	130	Brio, St.Jude	6148
14	1.9 mA	360	50	1.9 mA	360	50	RC Activa, Medtronic	3389
15	1.5 mA	208	190	1.5 mA	208	190	Libra, St.Jude	6148
16	1.5 mA	180	100	1.6 mA	180	100	RC Activa, Medtronic	3389
17	1.8 V	360	60	1.8 V	360	60	RC Activa, Medtronic	3389
18	1.4 mA	212	130	1.5 mA	212	130	Brio, St.Jude	6148
19	1.8 V	180	130	1.6 V	180	130	RC Activa, Medtronic	3389
20	2.2 V	240	60	2.2 V	240	60	Kinetra, Medtronic	3389
21	1.8 V	180	130	1.8 V	180	130	RC Activa, Medtronic	3389
22	1.4 V	300	130	1.4 V	300	130	Kinetra, Medtronic	3389

Amplitude is in [V], if voltage mode of DBS was used; amplitude is in [mA], if current mode of DBS was used.

### Clinical assessment

The severity of clinical improvement was assessed in the beginning of each session with scales recommended for dystonia^61^. Patients with generalized dystonia were evaluated by using the motor part of the Burke-Fahn-Marsden Dystonia rating Scale (BFMDS – before implantation 36 ± 20, BFMDS – after implantation: DBS ON 26±23, DBS OFF 30±23) and patients with cervical dystonia by using the severity score of the Toronto Western Spasmodic Torticollis Rating Scale (TWSTRS – before implantation 25 ± 3; TWSTRS – after implantation DBS ON 15 ± 7, DBS OFF 19 ± 3). The long-term benefit of GPi DBS treatment was expressed as a relative change in the adjusted dystonic score (BFMDS or TWSTRS) between the GPi DBS ON and the preoperative state. To check whether clinical improvement depends on the duration of chronic DBS, a correlation of the interval elapsed from the DBS surgery with the relative preoperative/postoperative change of the adjusted dystonic score and with the GPi DBS ON/OFF relative change of the adjusted dystonic score were analysed.

Patients with a clinical improvement >50% were considered as good responders, patients with an improvement of 25–50% as partial responders and patients with improvement <25% as non-responders.

### TMS

Each subject was examined in sitting position with the chin and forehead held by a supporting frame and with the arms freely positioned on the legs. TMS was performed by using a magnetic stimulator BiStim (Magstim Co., Dyfed, UK) with the figure-of-an-eight-shaped coil. The handle of the coil was pointing posteriorly and laterally ~45° to the sagittal midline of the subject’s head. The stereotactic navigation system (Brainsight Frameless, Roque Research, Canada) was used to ensure the localization and reproducibility of the “hot spot” between sessions.

Magnetic pulses were delivered to the optimal position above the primary motor cortex for producing a motor evoked potential (MEP) in the Abductor Pollicis Brevis muscle (APB) contralateral to the position of the coil. Simultaneously, the MEP was recorded from the Abductor Digiti Minimi muscle (ADM). Surface electromyografic (EMG) electrodes with a belly tendon montage were used to record the MEP from both muscles. The native EMG signal was amplified and filtered in a band-pass from 5 Hz to 2 kHz (CED 1902 Quad-system amplifier, Cambridge, UK).

TMS of the motor cortex is considered to be a safe procedure in patients with electrodes implanted for DBS^22,62,63^. To minimize the risk, a safety distance of at least 20 cm between the implanted pulse generator and the TMS coil was kept.

#### Motor threshold

The resting motor threshold (RMT) was defined as the minimum TMS intensity that produced a liminal MEP (about 50 μV in 50% of ten trials) at rest. The active motor threshold (AMT) was defined as the minimum stimulus intensity that produced a MEP of about 200 μV in 50% of ten trials during isometric contraction of the tested muscle at 10% maximum.

#### Short-latency intracortical inhibition (SICI)

A paired pulse TMS protocol by Kujirai^18^ was employed using a subthreshold conditioning stimulus followed by a supratreshold testing stimulus with an interstimulation interval of 2.5 ms. Three consecutive measurement blocks, each consisting of 15 paired TMS pulses randomly intermixed with 15 single TMS pulses, were recorded in each session. The intensity of the conditioning stimulus was set to 70% of the AMT in the first block, to 80% of the AMT in the second block and to 90% of the AMT in the third block. The intensity of the testing TMS stimulus was always set to 130% of the RMT. In each block, the peak-to-peak amplitude was measured separately on the averaged MEP elicited by the paired pulse TMS and on the averaged MEP elicited by the single pulse TMS. The SICI was then expressed as a percentage of the unconditioned MEP.

#### MEP onset latency

The assessment was done manually by experienced rater who was unaware of the actual DBS state. The MEP onset latency was measured between the testing stimulus to the beginning of the initial deflection of the MEP on each of the 15 individual sweeps elicited by paired TMS pulses and 15 individual sweeps elicited by single TMS pulses in each of the three blocks. The values were then averaged, providing one mean value for the single pulse MEP and three mean values for the paired pulse MEP considering three different AMT intensities of the conditioning stimulus separately. This was done independently for each of the two GPi DBS states.

#### Spontaneous muscle activity

Each subject was asked to keep all hand muscles relaxed during the paired pulse TMS experiment. To ensure that the muscle tone of the APB and ADM was not affected by underlying dystonic activity and that spontaneous activity remained stable in the GPi DBS OFF and ON states, a rectified EMG was analysed on both muscles during two intervals within each individual sweep in each patient throughout the study: (i) in the interval starting - 10 ms before the TMS pulse and lasting 10 ms and (ii) in the interval starting 80 ms after the TMS pulse and lasting 20 ms, because both of these intervals were free of the stimulus artifact or MEP. According to previous studies^64,65^ we calculated a threshold of pre-activated muscle activity defined in each patient as the mean ±3 SD amplitude of the spontaneous EMG activity determined separately for the GPi DBS OFF and ON states. Then we calculated the number of sweeps exceeding this threshold. Additionally, we calculated the area under curve of the rectified EMG in each of the two intervals for both muscles for each DBS state.

### MRI

Imaging was performed using a 1.5 T MR scanner (Siemens Symphony; Erlangen, Germany) with a T1-weighted magnetization prepared rapid acquisition gradient echo (MP-RAGE) sequence. Structural images were acquired with 176 sagital slices using a nominal resolution of 1 × 1 × 1 mm^3^ and FOV 238 mm covering the entire brain and cerebellum (TR = 2060 ms; TE = 3.93 ms; flip angle = degrees; TI = 1100 ms). Imaging was performed according to previously defined technical precautions considering the potential hazard in patients with intracerebral electrodes^66,67^. Image pre-processing was performed using the CAT12 toolbox (www.neuro.uni-jena.de/cat12) and SPM12 (www.fil.ion.ucl.ac.uk/spm) with MATLAB 2016b (MathWorks, Nattick, MA). The T1-weighted images were spatially normalized to MNI space using the DARTEL algorithm^68^. To account for volume changes during normalization, GM density in each voxel was then modulated by the amount of non-linear deformation. Finally, the scans were smoothed with a smoothing kernel of 8 mm full-width at half-maximum (FWHM).

### Statistical analysis

Native data (SICI and MEP onset latency) were logarithmically transformed to achieve assumptions for further used methods. The SICI and MEP onset latency were analysed using linear mixed effects regression models with fixed factors: *‘DBS state’, ‘intensity’ of the conditioning stimulus, ‘muscle’, ‘benefit’ and ‘dystonia type’*. As the effect of ‘*dystonia type’* was found as nonsignificant, this factor was omitted in the final models. The pair of the patient and his/her age-matched healthy control was considered as a random effect. The R statistical package version 3.2.3 (R Foundation for Statistical Computing, Vienna, Austria, www.R-project.org) was used for statistical analysis. P-values less than 0.05 were considered as statistically significant.

Voxel based morphometry was performed using a general linear model controlled for age and total intracranial volume as confounding factors. The differences in GM density between healthy controls and dystonia patients were tested with ANOVA approach with benefit of the GPi DBS therapy as independent factor. The GM density was correlated with the mean SICI effect (average of 70%, 80% and 90% AMT of the conditioning TMS stimulus, average of both the APB and ADM muscles regardless to GPi DBS state) and third, the GM density was correlated with the MEP onset latency after single and paired TMS pulse (average of 70%, 80% and 90% of the AMT of the conditioning stimulus, the APB muscle in OFF state).

In all analyses, a cylinder-shaped mask with a diameter of 30-mm around each electrode was used to exclude tissue affected by susceptibility artifacts generated by implanted electrodes. In analyses with fixed factors, family-wise error (FWE) correction was applied at the voxel-level with p < 0.05. The correlation analyses were corrected less conservatively using an uncorrected voxel-threshold of p < 0.001 with FWE correction at the cluster level with a threshold of p < 0.05.

The area under curve and the number of sweeps in which spontaneous muscle activity exceed the individual threshold was compared for both analysed intervals and both GPi DBS states using the Wilcoxon signed-rank test.

## Electronic supplementary material

Supplementary figure

## Data Availability

The datasets analysed during the current study are available from the corresponding author on reasonable request.
