# Supplementary material for: Benefits of pallidal stimulation in dystonia are linked to cerebellar volume and cortical inhibition
Source: Sci Rep. 2018 Nov 21;8:17218. doi: 10.1038/s41598-018-34880-z (PMC6249276; doi:10.1038/s41598-018-34880-z)

**Supplementary information for:**

**Benefits of pallidal stimulation in dystonia are linked to cerebellar volume and cortical inhibition**

Anna Fečíková^1^, Robert Jech^1^, Václav Čejka^1, 2^, Václav Čapek^1^, Daniela Šťastná^3^, Ivana Štětkářová^4^, Karsten Mueller^5^, Matthias L. Schroeter^5,6^, Filip Růžička^1^ & Dušan Urgošík^7^

^1^Department of Neurology and Centre of Clinical Neuroscience, First Faculty of Medicine and General University Hospital, Charles University, Prague, Czech Republic

^2^Faculty of Biomedical Engineering, Czech Technical University in Prague, Czech Republic

^3^Department of Neurosurgery, Na Homolce Hospital, Prague, Czech Republic

^4^Department of Neurology, Third Faculty of Medicine, Charles University and Faculty Hospital Kralovske Vinohrady, Prague, Czech Republic

^5^Max Planck Institute for Human Cognitive and Brain Sciences, Leipzig, Germany

^6^Clinic for Cognitive Neurology, University Hospital, Leipzig, Germany

^7^Department of Stereotactic and Radiation Neurosurgery, Na Homolce Hospital, Prague, Czech Republic

**Supplementary Figure 1**

Figure shows a single motor evoked potential (MEP) obtained from the APB muscle of patient #10 in the relaxed state elicited by the single pulse TMS (See Figure 1 A) and by the paired pulse TMS (see Figure 1 B) during the GPi DBS OFF and ON. The MEP onset latency was measured between the TMS pulse and initial deflection of the MEP on each of the individual sweeps.


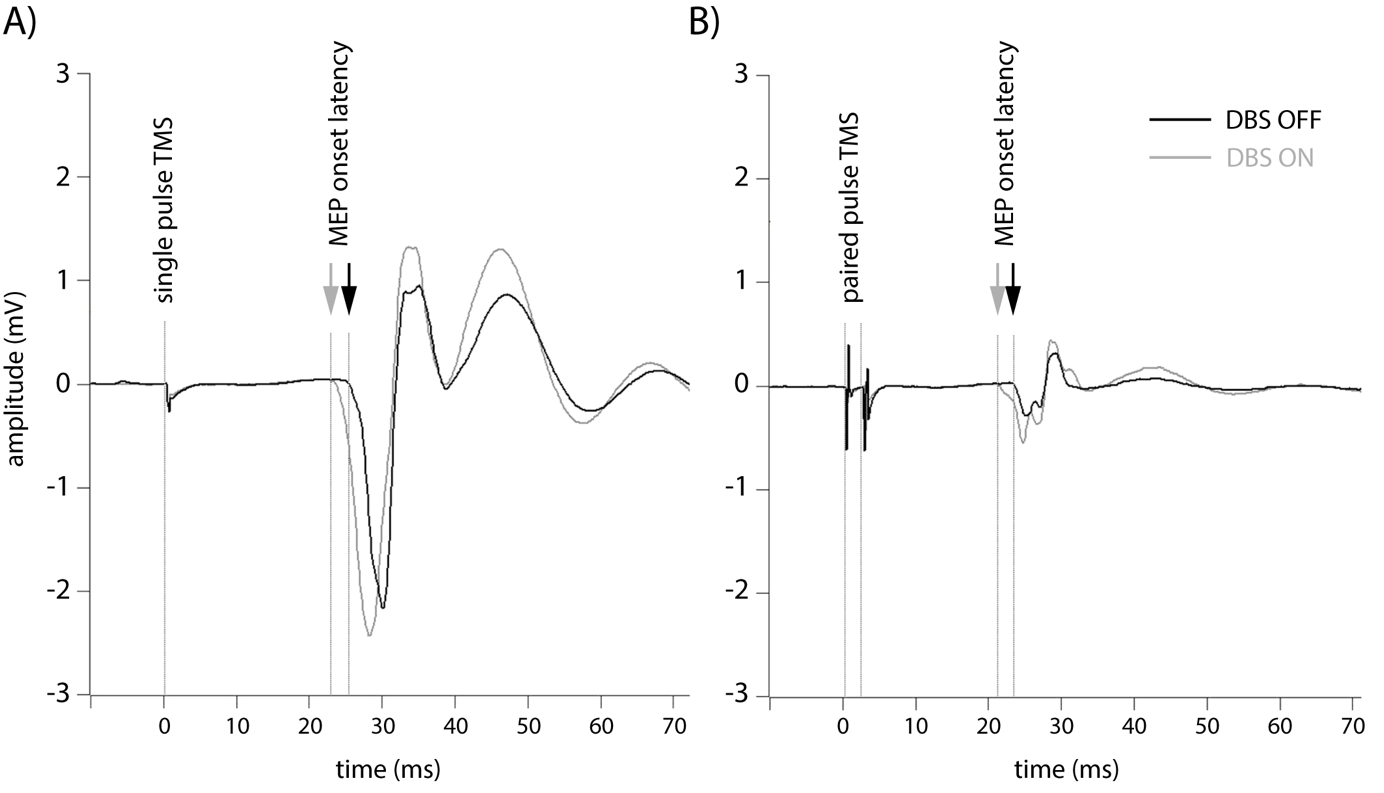

Supplement: Supplementary file 1 — Supplementary figure [file 41598_2018_34880_MOESM1_ESM.docx]
